# Erasmus students’ motivations in motion: understanding super-mobility in higher education

**DOI:** 10.1007/s10734-022-00852-6

**Published:** 2022-04-23

**Authors:** Valentina Cuzzocrea, Ewa Krzaklewska

**Affiliations:** 1 Department of Political and Social Sciences, Università Degli Studi Di Cagliari, Cagliari, Italy; 2 Institute of Sociology, Jagiellonian University in Krakow, Krakow, Poland

**Keywords:** Higher education, Study abroad motivations, Erasmus +, Spatial reflexivity, Super-mobility

## Abstract

This paper looks at youth mobility as an ongoing, dynamic, processual experience in the making of an educational trajectory. By exploring the experiences of students who have undertaken more than one mobility experience under the Erasmus + program, we reflect on how underlying motivations change over the course of subsequent mobility experiences. In contrast to existing research, where the focus has been on reported motivations for one-off mobility experience, we discuss the latent motivations driving super-mobile educational trajectories. In doing so, we observe the ongoing reconfiguration of these trajectories through the concept of spatial reflexivity, which results in articulated and augmentative dynamics over time. Methodologically, the paper is based on qualitative material collected in person and online with such mobile young people across Europe.

## Introduction

Developments within higher education urge us to look at fragmented, accelerated, and multiple experiences of mobility beside one-off stays abroad (Czerska-Shaw & Krzaklewska, 2021a; Cairns, 2021; Robertson et al. 2018). This focus is especially relevant in the case of students using mobility programs as much as they can during the time spent in higher education. Looking at higher education students who have undertaken more than one mobility experience under the Erasmus + program, we investigate intensive mobility with higher education, with particular attention to underlying motivations to repeat the experience of mobility, up until eventually engaging in “super-mobility”.^1^ Within this study, our interest is specifically on opportunities enacted and activated at an individual level, rather than institutionalized “super-mobility,” where students participate in a university designed mobility-intensive study program (Czerska-Shaw & Krzaklewska, 2021a). In the current study, the “self-made” super-mobile subject constructs his/her educational trajectory through a mixture of institutionalized and non-institutionalized opportunities for short-term stays abroad, depending on individual creativity, adaptability, and ambition, somehow in alignment with the notion of the entrepreneurial self (Kelly, 2006; Oinonen, 2018).

While most students will never experience transnational mobility during education, a certain number seize such opportunities, and there will be those who seem to grasp every single opportunity on offer. Under 2018 Erasmus + call, 350,000 students undertook studies or an internship (European Commission 2018). Structural opportunities are critical, as we observe a richness in mobility programs across the EU, but most of all the possibility within Erasmus + to go for study abroad multiple times in various forms. The Erasmus program was founded in 1989, and it currently remains the most well-known one in Europe. It is unique for being linked to the political project of the EU, as well as for sustaining a specific vision of the mobile European citizen as a cosmopolitan mobile worker (Recchi 2015; Feyen & Krzaklewska, 2013; Cairns et al., 2018). Within this program, higher education students spend one or two semesters abroad in an educational institution with whom their home university has a cooperation agreement, mostly in Europe, but also beyond. Additionally, the program provides students and recent graduates with the opportunity of undertaking internships abroad.

With the launch of Erasmus + edition of the program in 2014, the prospect of students developing a super-mobile trajectory during their academic studies lies in the possibility of spending up to 12 months of Erasmus mobility at each level of their studies (BA, MA, PhD). No official statistical data exist on the actual trends in multiple usage of the program, and no substantial research has been undertaken to explore the uptake of these opportunities. Therefore, little is known about the differentiation between one-time or multiple users. Indeed, analyzing separated episodes without this differentiation in mind prevents us from capturing their meaning within a broader educational trajectory (Cairns, 2021).

We conducted 18 in-depth interviews with students or recent graduates from 9 countries, who had completed more than one stay abroad within the program. Our approach in this article differs from other studies on Erasmus students’ motivations which have concentrated, in most cases, on declared reasons for singular studying abroad episodes, often using quantitative methods. This list usually includes personal development, having new experiences, language learning, cultural learning, meeting new people, academic enhancement, living outside one’s country, improved employment perspectives, and gaining independence (Aresi et al., 2018; Krzaklewska, 2008; Lesjak et al., 2015).

On top, gaining experience abroad was stressed by one of the pioneering in-depth studies in the field (Murphy-Lejeune, 2002). We follow this approach, which underlines wider institutional, cultural, or structural contexts for mobility, called “latent” motivations (Murphy Lejeune 2002). However, taking an agentic and dynamic perspective, we also investigate how the motivations for stays abroad transform over the course of multiple mobility experiences, as individuals become increasingly confident in their abilities and social skills for transforming their present and future (Tran and Vu 2018). Looking at how “zones of opportunities” are sought after allows us to highlight the process of realization of personal ambitions vis-à-vis structural conditions available. For a study centered on an EU-based program, this is especially important for so-called European peripheries (Marcu, 2019), where resources to draw on to construct successful professional paths may be limited. Theoretically, building upon the concept of spatial reflexivity by David Cairns (2014) and reflections around young people’s temporalities (Brannen & Nilsen, 2002; Cook, 2018; du Bois Reymond, 1998; Leccardi, 2005), we consider the dynamics within which to frame motivations for mobility. These motivations are themselves “in motion”: they are in fact being constantly reinterpreted at the different stages of education with attention to broadening possibilities for a future career. We also attempt to link research on higher education students’ mobility with key debates on the growing reference to the spatial dimension where youth transitions take place, the normativity that comes with it, and its perceived potential for personal socio-economic advancement (Cairns, 2014; Farrugia, 2016; Robertson et al. 2018; Yoon, 2014; Tran and Vu 2018; Nikunen and Ikonen 2021).

## Time and space in educational trajectories

Higher education systems across Europe actively promote mobile trajectories within institutional networks (Cairns et al., 2017); the “mobility imperative” (Farrugia, 2016) is strongly present in the academic milieu (Courtois, 2020). We refer here to pre-pandemic conditions, with networks of institutions constituting “paths” for mobility and institutions, in particular the European Union, based on extensive funding. It is in this context that the notion of the “super-mobile” student emerges, for whom education appears to occur “on the move,” with growing opportunities for repeated mobility experiences during one’s education. Such notion arises through the frame of a neoliberal university system and responds to the expectations that students need to differentiate themselves through ambitious curricula and excellent performance or—pointedly—distinctive mobility patterns, including exotic locations and ambitious study programs (Hof, 2019; Prazeres, 2019). Within those institutional set ups, then, some students experience learning as a phenomenon that happens *throughout* spaces—with their educational programs being detached from a physical space or singular institution. Time spent abroad is regarded as an effective tool for learning, and such learning might be reconceptualized as not exclusively related to the “academic” sphere (Krzaklewska, 2013; Yoon, 2014), with students being aware of the necessity to cumulate a baggage of experiences, also of non-formal character, that is meant to make their curriculum attractive to future employers.

Since the Bologna Process came into effect in 1999,^2^ the international organizational warps have become ever closer, imposing a process of rationalization on both institutions and students alike, even if not without criticism (Oinonen, 2018). At the same time, ironically, the Bologna Process was a turning point for thinking about student agency in education, given that educational choices have become more navigable, albeit more strictly time-bounded. Ultimately, students are invited to “play” within the system and navigate study tracks among proliferating options, many of which are of international character.

We use the concept of “spatial reflexivity” (Cairns, 2014; Cairns et al., 2012) as a starting point for an overarching theoretical framework in our analysis, as it helps us in defining the significance of opportunities for mobility. Spatial reflexivity is defined as “the incorporation of a geographical dimension into the transition to adulthood” (Cairns, 2014: 6), here in particular in their educational trajectories, but with a view on the expected labor market entrance. Students are made responsible for pursuing opportunities to study abroad by applying to programs and choosing a preferred destination out of those within their university network. Therefore, the choice itself is in a sense strategic, pondered at the crossroads of preferences and available opportunities. Spatial reflexivity links “spatial movement with socio-economic self-advancement” (Cairns, 2014: 6), a general claim that may in part be present among Erasmus students. And in fact the limitedness of the mobility options among which prospective Erasmus students are able to choose is the first structuring point that may perpetuate inequalities—as usually students from less prestigious universities may travel to similar level institutions, and the “fit” of mobility experience to their study program is often weak (Courtois, 2018).

Additionally, we claim it is necessary to incorporate a temporal element into this frame. This pertains to the fact that students are aware that their time for institutionally supported mobility is limited to the duration of their studies. Much of youth research has portrayed “the time of youth” as a specific, finite period in one’s biography (Cavalli & Calabrò, 1985). Biographic time works as a bridge between the experience of biographical space and a broader temporal frameworks (Leccardi, 2009). Youth thus is socially envisioned as a period of time where obligations are suspended (Brannen & Nilsen, 2002), and it is socially acceptable to take time to explore (du Bois-Reymond 1998).

The limitedness of this particular biographical moment for mobility in education is important to bear in mind when analyzing super-mobile students. Indeed, such young people may be exceptionally good masters of their time, making the most of it, whatever the plan they are pursuing *while* in education. This partially contrasts with debates pointing to a lack of projectuality as a constitutive trait of young people, with young people concentrating on living in the present and producing narratives that enable them to avoid thinking about the future (Leccardi, 2009). After Compton-Lilly (2016), who stresses the importance of reflexivity during time spent in education, we assume that reflections on time are key to nurture motivations of mobility. In sum, we contend that it is necessary to consider both temporal and spatial reflexivity in the construction of an educational trajectory. Education happens through a finite, compacted time, while learning occurs throughout a multiplicity of spaces. Considering how these layers converge is a key contribution of this article.

## Methods

The focus of this article is on super-mobile university students. With this in mind, in 2017–2018, we conducted in-depth interviews with 18 students or recent graduates who had completed more than one stay abroad within the Erasmus + program. This comprised of at least one study exchange, accompanied by at least another study exchange or an Erasmus internship. The participants, 14 females and 4 males, came from diverse faculties, mainly socio-political sciences, economy, literature, psychology, or language, but also from architecture, physiotherapy, engineering, or mathematics. They were between 21 and 29 years of age and from 9 countries, with either a long-standing membership in the EU (Italy, Austria, Germany, Luxembourg), “new” member states (Poland, Slovakia, Romania), or candidate country (Turkey), plus Russian Federation. The drawbacks of our self-selected sample relate to a lack of gender balance and low representation of STEM faculties (which reflect the program) and mild overrepresentation of Polish respondents (7). Also, the reliance of former Erasmus student networks in recruitment might have caused a bias towards more active students. Nevertheless, the resulting data is rich and provides a unique insight into the experiences of this understudied group, allowing us to take into consideration a variety of educational systems and labor market conditions.

The focus of the interview guide was on comparing subsequent mobility experiences in relation to motivation and included looking at activities, challenges, outcomes, and ways of developing and maintaining social bonds. The interview guide encouraged participants to create a narrative around their mobility project. The retrospective approach is fruitful in relation to studying mobility trajectories, as students seem to be expressing the wholesomeness of the experience abroad, rather than the pre-departure motivations of each move (Murphy-Lejeune 2002). The analysis was of inductive character with a support of the computer software for qualitative analysis of data. On the one hand, we analyzed each case scrutinizing the development of educational trajectories and the presence of mobility episodes at its different stages; on the other hand, we compared and contrasted cases between themselves (Miles and Huberman 1994). The challenge for researchers is how to capture the dynamic of motivations in motion, which may be numerous and intertwined but all encapsulated within the broad imaginative frames of learning and development that happen abroad.

## Motivations “in motion”: towards reflexive super-mobility

In this section, we analyze the interview material drawing from Cairns’ work on spatial reflexivity (2014) as a tool that enables us to see how motivations for mobility change in time, becoming less conformist and more reflexive. Some students had initial views on the program itself that would have developed later on. Regardless of how these changed, it was not uncommon to assume from the start of the university studies that they would have participated at some point, which indicates a normalization pattern (Courtois, 2020) that views Erasmus as anything “out of the ordinary”:

It is not that you say you went to Vilnius or Portugal and people say ‘Wow’, people say ‘Ok’. They all already were there. So Erasmus is now more popular, more people do it, because it is just a usual thing to do. (Marzena, female, Polish, aged 26, Erasmus in Lithuania and Portugal)

Indeed, enrolling to university is thought in conjunction with the possibility to embark on an Erasmus, which is part of an educational plan. “Erasmus for me was clear from the beginning when I started to study that I’m going to go abroad with Erasmus,” stated Isana, who visited Belgium and Poland from Austria. In the narratives of mobility that we collected, the mobility trajectory usually started with an Erasmus study abroad during a Bachelor study, followed by a second study period (during Master or the same Bachelor study if the first study period was not full year) or a traineeship. A few students took an additional opportunity of study outside Europe. On top of these experiences, students reported participation also in short-term mobility episodes such as volunteer work or other forms of training, suggesting that exploitation of mobility opportunities take place besides Erasmus too.

Admittedly, some initial experiences came about by chance or circumstance (“it wasn’t my decision, it wasn’t my plan”), or as a result of imitating their peers (“they applied, so did I”), which links to the peer relations’ impact on studying abroad (Beech, 2015). For instance, Gaye, a Turkish student, reported to us:

After my first experience, when I was back home, I decided that I’m definitely going for the second time, but before going on Erasmus, even though I was always together with Erasmus students and I was encouraged enough, I wasn’t really sure if I want, you know, spend my summer in another country but I think it’s all about experience: once you get it, you don’t want to stop it. And, this is definitely true for Erasmus. (Gaye, female, Turkish, aged 23, Erasmus in the Netherlands and Germany)

Only in the course of the experience plans were developed: the first Erasmus stay constitutes a trigger for continued, intensive mobility.

Regarding the choice of destination, many participants recalled an initial dream-like—which links to the less rational side of mobility decision-making (Cairns et al., 2018). An Italian student interviewed was able to nurture her love for the Balkans through repetitions of Erasmus visits there similarly for another interviewee who was mainly fascinated by Spain. Again, the Turkish student already mentioned seemed to make her final decision based on what her heart was “telling” her to do, without rationalizing much:

It was a hard choice for me, because the Netherlands was the destination that I was always dreaming of for years, like, from my plan, but at the same time when it comes to the city I realized that I would have be more happy in Helsinki than in a small city in the Netherlands (…) in the end I just followed my heart. (Gaye, female, Turkish, aged 23, Erasmus in the Netherlands and Germany)

We note that while some students might have initially been a little conformist and naïve in their mobility intentions, for instance by assuming they would overcome easily linguistic issues, a broader reflexive project tends to flourish as the time passes.

Eventually, experiences of mobility were transformed into a sophisticated and articulated performance of the (mobile) self. Subsequent stays were presented in markedly different terms as a result of this reflexive process and were embedded in increasingly pragmatic strategies, particularly in relation to one’s potential professional career (Tran, 2016). Even in cases where students used the program as an opportunity to devote time to having fun and socializing, subsequent experiences were still portrayed in rational terms as sound biographical choices (Yoon, 2014), as the case of a Polish student who ascribes particular aims to each mobility period:

Everything started in Hungary [first Erasmus], I really experienced the offer of Erasmus, and it motivated me to go to other countries, when there is a possibility.(…) Like a trigger. (…) my every Erasmus focused on another part. Erasmus in Hungary focused on social parts, integration. Erasmus Mundus in Brazil on travelling, and Erasmus in Germany on academic part (…)

And are you planning also to be like, moving in different countries, or travelling, or being mobile in your future?

Yes. Sure. I like travelling, and maybe I will go as a PhD to some scientific conferences in other countries. Yes. I think that I will keep going on my international experiences. (Maciej, male, Polish, aged 25, Erasmus in Hungary, Brazil and Germany)

Here, we see motivations for super-mobility strengthening, and, in fact, in this narrative, each consecutive stay clearly builds upon the previous experience. As time unfolds, we observe a process of appreciating the options that seem more and more available and real. The same goes for students’ awareness of structural barriers, in consideration of competences and passions, but also limitations:

I was less prepared for the first Erasmus and I had a totally different expectations and I was leaving with a lot of fear. Whereas during my second one, I already knew what to expect, I took care a lot of more about my language before and I was quite, I wasn’t nervous, I wasn’t scared that I wouldn’t meet anyone or that I would feel lonely. (Kasia, female, Polish, aged 25, Erasmus in Spain and Italy)

As in the case of the student quoted above, respondents themselves realize their maturation process, when they critically evaluate their own attitudes change. A male student from Turkey, for instance, insisted on going to Poland in search of intercultural experiences despite his national university system disincentives repetitive departures, which seems to us a strong affirmation of his plan:

And why I wanted to go Poland again or Erasmus again? The first thing, this freedom, this self-confidence is a cultural learning point. It affects me a lot and I turn back to my country. I felt totally different and I started to look around for international people. It’s like water for me, okay? (Ozge, male, Turkish, aged 27, Erasmus in Poland twice and Portugal, our italics)

As time goes by and students become experts in mobility, they get familiar with sources, rules, and administration of funding; discuss with us the technicalities of this; and show to be increasingly aware of how to use opportunities to give shape to an educational trajectory. In other words, they accumulate mobility capital (Murphy-Lejeune, 2002) that constitutes a basis for further geographical movement, as shown by Carla:

I did this experience in Portugal [third Erasmus], actually it was the best one among the three, and I think it was the best one because I did Erasmus before, so I knew how to take advantage of the situation and my language skills were also better. I think I take advantage because of the first two experiences, so that’s how I knew how to enjoy my stay in Portugal. (Carla, female, Italian, aged 26, Erasmus twice in France and Portugal)

The view of another participant eloquently sums up this section, declaring that, “[My] first Erasmus was like the seed was planted” (Magda, female, Polish, aged 24, Erasmus in Czech Republic and Spain). Many had not initially planned that they would have engaged in mobility while in education to such extent; diverse circumstances determine a development towards an eventual super-mobile career. On top of these general trends in how the motivations unfolded, we observed three latent motivations of mobility that fitted a reflexive project of the self, involving both spatial and temporal considerations: achieving distinction, developing the self, and living youth fully. We now turn to illustrate them.

## Achieving distinction

The first latent motivation is achieving distinction. While most student trajectories developed unplanned, as time passes, students realize the potential that lies in mobility for constructing a unique super-mobile educational trajectory. The necessity to distinguish oneself (Hof, 2019; Prazeres, 2019) is aligned with a neoliberal discourse of excellence, based on the principle of the entrepreneurial self and higher education competition. Given the normalization of going abroad and its massification (Courtois, 2018), and a matching discourse on aspiration (Nikunen and Ikonen 2021), doing only one Erasmus might seem like nothing special, as one should strive to pointedly differentiate from others. Indeed, our respondents were well aware of the strengths and weaknesses of their CVs. The more mobility experiences they acquired, the more reflexively aware they became of what they should do in order to increase their employability, particularly in the terrain of a European labor market, or else where they should go to distinguish themselves (Courtois, 2020; Hof, 2019).

It was good to do them both, I had two different experiences. I also think about my CV… this may be stupid… I think it is very nice, and if my future supervisor sees it… this is something I am proud of! My CV looks super good. Even if I suffered for some time, it has more benefits. (…) (Isana, female, Austrian, aged 23, Erasmus in Belgium and Poland)

With subsequent mobility experiences, additional factors beyond quantity of experiences were considered a way to achieve distinction, such as a wider array of locations, but also other factors, e.g., the prestige of universities. One of respondents started with a safe-mode mobility (going to a close-by country to do some travels), and later undertook an internship in Spain to gain professional experience, to finally join a more academically aimed study period at a selected institution:

I know that Granada University is like not the best in Spain but it’s like in the top of the list, so I want to learn. I want to write my Master’s thesis and I want to come back with some research there in Granada about [mentions topic]. (Magda, female, Polish, aged 24, Erasmus in Czech Republic and Spain)

Expressions used often related to doing “something more,” as exemplified by a Polish female student who expressed her motivations in relation to distinction-making, a pattern characteristic for Eastern European countries (Hemming et al., 2019). As her story unfolded, she told how she had undertaken multiple stays abroad to build her professional profile and differentiate herself from her peers to end up studying at a program in the USA:

And I was ready and brave enough to do it, so I took that step and I went to the US [work&travel programme]. When I was there, I was dreaming already about another Erasmus because that was a really big compliment in my studies. I want to come back to [the U.S] to do another degree, but also Erasmus encouraged me to do it. Because I knew that if I came back to Poland, that might be a little bit boring and I would be the same as everybody. I wanted to do something different. That’s why I wanted to apply for more Erasmus to make the best out of my studies. (Sylwia, female, Polish, aged 23, Erasmus in France, Bulgaria and Portugal)

One important spatial decision concerns the choice of destinations. Most students tended to differentiate the countries they go to, as one of them stated: “I’m not going to spend 10 months in one place but I’m going to spend one year in three different ones.” In opting for a plurality of experiences, and with confidence growing in the process, they orientated themselves to out-of-Europe experiences, often occurring as late mobility episodes, sometimes after the third mobility experience. Only in the case of two language-faculty students all mobility episodes happened in the same country, but this indicates an investment into a specific language-focused trajectory more than anything else.

However, as it emerges clearly in our sample, destination choice is also linked to an attractiveness of Western destinations to those coming from Central, Eastern, and Southern Europe (Hemming et al., 2019; Tejerina, 2019; Cairns, 2014). Our empirical material interestingly suggests that self-positioning in this geography reflects, indeed, wide structural dynamics. Southern and Western countries are popular destinations in general, while Central-Eastern European countries are often chosen because they are perceived as safe or easy to reach, or a cheap option, but also because it is “original” (Krzaklewska et al., 2021).

We know from previous studies that the payoffs from the stay abroad remain higher for students from, e.g., Southern regions (d’Hombres & Schnepf, 2021). However, in several interviews, we are able to see how a newly perceived need to distinguish oneself developed precisely through the mobility process; it became refined within a relatively limited period of time, fed by the ability to recognize the possibility of integrating existing abilities with complementary ones and also how to obtain them. As typical from our sample, a respondent from Austria seems to play on the time-frame allowed going to two different countries for a semester in each, instead of spending 1 year in a singular institution, because she “wanted to make more out of this experience.”

Unpredictability is also embedded somehow in such trajectories, allowing for learning how to capitalize newly obtained resources. Another student was admitted to a very prestigious university in France, of which she was not aware at the moment of applying. Only afterwards, she realized she could exploit this experience to apply to the best universities in the USA. Her first exchange in France was quite a challenging one socially; this is why she decided to prolong her studies and have a second—more successful—try for an Erasmus experience in Spain. This led to the decision to spend 1 more year working in Spain to then target the USA:

I decided to go to the United States before I decided to go for my Erasmus experience. But it will profit my application, because of the Erasmus experience I know two more languages. I’ve lived in two different countries. I’ve been to one of the best political science universities in Europe, so, only profits from this exchange. (Agnieszka, female, Polish, aged 23, Erasmus in France and Spain)

## Developing the self

The second latent motivation is aligned with a learning-oriented attitude which fits into a developmental project of the self. This dimension is not exclusively related to the employment sphere and has a lot to do with a fundamental need to grow as a person. This may be linked to the concept of aspirations (Nikunen & Ikonen, 2021), whereby self-development becomes an agenda for the self and mobility is seen as a space for self-exploration and identity reflection (Cuzzocrea & Mandich, 2016). Studies on international student mobility have highlighted mobility as a medium for “becoming the kind of person [one] want[s] to be” (Tran, 2016). Even if clearly entangled within the employability discourse (Yoon, 2014), this aspect is apparent in the narratives of the respondents, highlighting the growing need to be aware of one’s self-development and to assure mobility as space for such explorations.

And it really changed my view and this is why I also see myself as different than my classmates (…) now I’m graduated but I’m planning to move to Germany, for example, and I realized that after my first Erasmus (…) I was let’s say stronger and I was trusting myself more, that I can do more and also, I developed myself in my study as well, because during Erasmus, of course, Erasmus is nice when you’re having fun but I was also studying so much, trying to see how physiotherapy works in different countries, especially in the Netherlands.(…) when I was back home and people around me, especially the person that I have in my internship, they realize that I’m actually different, so they tried to show me other possible things that I can or I can’t do. (Gaye, female, Turkish, aged 23, Erasmus in Netherlands and Germany)

After the first one I became more independent and I mean I was more able to cope with myself and spent more time alone after France, (…) I became more tough after this experience. And after Spain, I became, I don’t know how to describe it: even more open (…) even more welcoming of all of the differences you can see in other people. (…) this is really positive way of thinking I gained after Spain, which after France I wasn’t that positive actually. (Agnieszka, female, Polish, aged 23, Erasmus in France and Spain)

Along this line, it is very interesting to mention Laura, who has “always loved to travel” and felt “different” in the small town she comes from, which she perceives “too narrow for her.” Laura spent almost 4 years in various mobility experiences, including with European Voluntary Service, Erasmus traineeship, Erasmus Globus, and as an au pair. This experience is the backbone on which she has built a refined ability to use institutional programs to explore what she is more interested in—which in her case is a particular geographical area (the Balkans). Late experiences in particular allow her to gain not only meaningful work skills, but also to nurture her passions, and gave her the confidence to pursue further goals also in private life.

Importantly, this latent motivation is linked to the need to recognize that learning is a multi-space experience which may occur across borders and different dimensions of life, as also supported by the European Commission (2009). Our participants defined a diverse range of learning mobility experiences in which they had been involved within and beyond the scope of traditional curricula, including exchanges, internships, intensive courses, festivals, and touristic endeavors. There was a view that when one is abroad, learning happens beyond the premises of a university or school, including the streets, through peer encounters, non-formal learning activities, student organizations or volunteering, and many other activities. To confirm this, several respondents were engaged in student organizations that supported exchange students at local universities and fed into their mobility-centered biography.

This motivation is also linked to the discourse on cosmopolitanism, intended as a vision of the self as a global citizen, an attitude of openness to other and to difference, to question spatial imaginaries and strive for intercultural encounters (Nowicka, 2012). Some participants wished to cultivate a cosmopolitan habitus over subsequent stays, ascribing to belong to a “Global Generation” seeing EU (and beyond) as open space to construct their lives (Pustulka & Winogrodzka, 2021). As in case of the quoted interviewee below, the first Erasmus led them to continue searching for more intercultural experiences—linked to international travel, companionship or future work environment, or participation in organization supporting Erasmus students. Ozge even founded an organization supporting incoming exchange students just after his second Erasmus. For Sylwia, the first Erasmus was about improving language, but the second was more directed at meeting people from different cultures:

Yes, I already have my languages at the level that I could communicate with the others because before that was also a big motivation to improve my languages and to practice it. And with the second ones, I think it was the willingness to still meet new people and to be connected to people with different background. Because if you are in your country, you’re excited to discover something new, I think. You become more curious. And I just didn’t want to get stuck in my current level and I still wanted to be surrounded with people from different countries. And travel and still keep on improving my languages. (Sylwia, female, Polish, aged 23, Erasmus in France, Bulgaria and Portugal, our emphasis)

Others used Erasmus as a test for their ability to migrate (Tran, 2016). The creation of international networks—mostly in the bubble form as in Cuzzocrea et al. (2021)—was clearly used as a source of support, identification, and bonds which allowed the aspiration to develop the self to become real.

## Living youth fully

A wide cultural discourse sustains the idea that youth is a phase of life that should not be “lost” (Ravert, 2009). Indeed, a third latent motivation for mobility derives from the perceived need to exploit mobility opportunities in youth. If the time devoted to education corresponds to a youthful phase of life, then explorations are limited to the period more or less from 19 years of age, to about 25–30 (depending on the norms within countries of origin). According to this (slightly evanescent, but somehow pressing) discourse, young people compare themselves to one another within a general peer group and do not want to be left behind in terms of life experiences (King, 2011). They want to be at least as successful as others, and being constantly on the move can be seen as to fulfill this expectation. Embodying this need, they show that they have learned how to better use Erasmus as a proxy for living their youth to the fullest. This has already been noted for other kinds of super mobile students who point to the importance of “experiencing something different” (Czerska-Shaw & Krzaklewska, 2021a), even going into the direction of a “consumeristic” approach to mobility (Yoon, 2014). In our study, there clearly was a pattern of seizing opportunities and grasping all potential occasions for mobility before the end of their educational trajectory:

[…] before the first Erasmus I have had my doubts but I went on Erasmus and it was great. So why not to try! So at least I have an impression that I would use all possible opportunities for mobility, all that exist at my university [laugh] All Erasmus, all that there are, and well exploit this exchange. (Anna, female, Polish, 24 years old, Erasmus twice in Spain)

The normative pressure around exploiting youth clearly emerged through the narratives, with students internalizing the value of mobility for young people global trajectories (Courtois, 2020; Farrugia, 2016; Yoon, 2014). Perhaps most notable were those who, having felt that they had missed out on the “real” Erasmus experience during their first exchange, later used the program to compensate for last opportunities. Reasons for Erasmus experiences failing to meet these expectations variably included a narrow focus, high academic excellence, personal difficulties, or isolation from other international students. Consequently, these were not considered to be “real Erasmus” experiences, in that such factors were seen to be preventing youthfulness from flourishing (Waters et al., 2011), rather than to enable it:

When I was in Belgium, I really felt that I couldn’t have the full Erasmus experience because the university was really, really tough,(…) I didn’t really have a lot of time to socialize because of all the assignments, and the assets that I had to do, it was a really a lot and I suffered a lot during that time. But now, when I look back at it, I feel that I gained so much more when it comes to the academic perspective. (Isana, female, Austrian, aged 23, Erasmus in Belgium and Poland)

Showing awareness about time constraints in their own biography, some participants wanted to exploit such opportunities before it was too late or simply take the chance when they felt they could. In this, mobility is seen as an experience that makes up part of the student life:

And basically, like I’m not getting any younger, so I know that it’s actually that my studies are coming to an end. So I thought, okay, I’m going to spend 6 years at the university instead of 5, so I want to get something out of it. I want to have the full Erasmus experience. (…) So I decided, okay, I’m not going to spend 10 months in one place but I’m going to spend one year in three different ones. (Magda, female, Polish, aged 24, Erasmus in Czech Republic and Spain)

Mobility experiences were strategically located in what they felt was the remaining time of youth, in an attempt to combine with personal lives and demands of the home universities (e.g., Master thesis, exams). Some even embarked on further university courses precisely because they had in mind this would have worked as a gateway to study abroad later on, but not too late. Interestingly so, even if time-bound, the super-mobility that is spread along the educational pathways as separate episodes does not bring to participants the impression of hurry or being in a rush—which was characteristic of institutionalized super-mobile students in the Erasmus Mundus program, who miss the opportunity to go back to their home country between destinations (Czerska-Shaw & Krzaklewska, 2021b).

## Conclusions

In this article, we use the concept of spatial reflexivity to develop a dynamic perspective on students’ super-mobility which also takes into consideration some temporal elements. Resultingly, by examining Erasmus students’ motivations as “in motion” not only in space but also in consideration of a biographical timing, we set the basis for disentangling important aspects of how a mobile educational trajectory is constructed. The discourses at stake in the decision to pursue multiple experiences of educational mobility, in the form of study exchanges or internships abroad, are linked to recent debates within mobility research. Our contribution elaborates on possible forms of super-mobility that are based on the perceived necessity to achieve distinction, develop the self, and live youth fully. “The more mobility, the better” might be a slogan to explain the motivations for mobility for our participants. Having accessed the system of mobility within educational systems, a desire for further travel grows, leading them to take various opportunities to utilize mobility—oftentimes pursuing (sometimes deliberately) more challenging contexts as a way to train themselves, to acquire competence, or to grow. Thus, mobility becomes a tool for learning, both in a traditional academic sense and also in terms of self-development and reflection. In subsequent mobility experiences, students are increasingly strategic in the pursuit of their educational goals, as well as aware of the pros and cons of their past choices, and thus more competent in the effective use of prospective mobilities. Notably, when making decisions on subsequent mobility moves, students not only decided *where* to go, but also *when* to go. The enactment of such decisions is a finding that contrasts with the assumption that youth have difficulties in planning their biographies and their future as a result of widespread uncertainty. In fact, our respondents are a clear exemplification of choice-biographies (du Bois Reymond, 1998), whereby students enact agency by expressing their initiative in modeling their educational trajectories outside standard patterns, sometimes even going against the normative expectations on, e.g., finishing degree on time. The super-mobility pace designed by students might also provide them with a unique sense of control over time, with new abilities to deal with deadlines for applications, departure frames structuring educational pathways and ultimately securing feeling of agency within available temporal structures.

In their awareness of the normalization of Erasmus mobility experiences, students took conscious decisions to differentiate themselves from their peers, fellow students, or other mobile students, as well as to draw a line between their past immobile self and a current lifestyle engaged in frequent dislocations (King, 2011). This shows how “spatial reflexivity” can enhance our understanding of dynamics of super-mobility, super-mobility being the ultimate, perfect product of it. Moreover, the anticipated time-frame available for educational mobility brings to the fore the ephemeral, evanescent, even fleeting social construction of youth; this is not an ever-lasting phase of life, and some of our respondents who were near to the completion of their educational trajectories started to explore their “final opportunities” for mobility even more fervently. The educational or learning process is by definition time bound; it has stages, contains a vision of progress, and occurs over time. Our respondents valued personal development, in particular regarding the aspect that it happened through a multi-space learning, and emphasized how they were able to excel through their experiences of mobilities (Nikunen and Ikonen 2021). Even in light of the current quest for life-long learning, the period of youth is still seen as best for educational and learning activities, and as such, they were keen to exploit it. While it may be debatable if it always provides a privileged position in transnational spaces (e.g., Brooks & Waters, 2011), in particular in times of the COVID-19 pandemic (Wang, 2021), student status remains a gateway to funding opportunities for mobility and ensures anchoring to a higher education system that gives them credibility.

## Footnotes

1 This happens when students “circulat[e] inside a network of institutional ties that operate in the spaces between traditional higher education structures” (Czerska-Shaw and Krzaklewska 2021a: 200).

2 The Bologna Process is an agreement between European countries, which aims to homogenize higher education to ensure comparability in the standards and quality of education. For instance, most study courses are standardized into 3-year undergraduate course (BA) and a postgraduate level 2-year MA course.
